# Supplementary figures and images for: Light triggers a network switch between circadian morning and evening oscillators controlling behaviour during daily temperature cycles
Source: PLoS Genet. 2022 Nov 11;18(11):e1010487. doi: 10.1371/journal.pgen.1010487 (PMC9683589; doi:10.1371/journal.pgen.1010487)

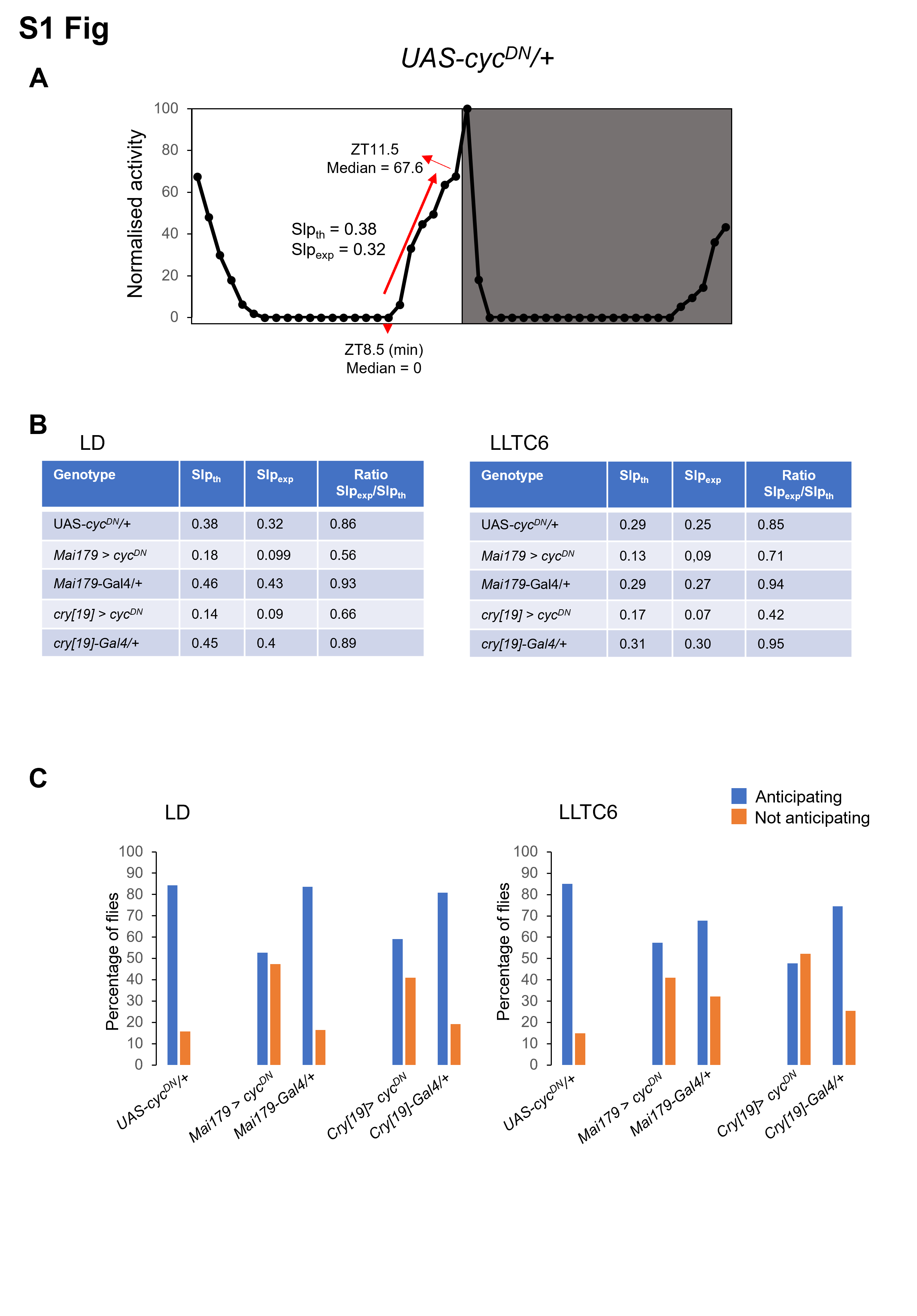

Supplement: S1 Fig — A) Representative median of the normalized activity of control flies in LD. The white rectangle delimits the 12h of light while the grey box delimits the12h of darkness. This example explains the calculation of the slope (theoretical, Slothe, and experimental, Sloexp). Slothe is calculated from the median levels according to the formula (ActZTmax- ActZTmin)/(tZTmax-tZTmin) explained in Materials and Methods. For example here, the median activity of the first maximum before light-off (ZT11.5) is 67.6. Hence, here Slothe = (67.6–0)/(691–511) = 0.38. The calculation is made with the ZT in minutes. Sloexp is the median of the individual calculated slopes. B) Values of the Slothe, Sloexp and the ratio Sloexp/Slothe of the indicated genotypes in LD and LLTC6. If fly activity is highly synchronized between individuals, the ratio of Sloexp/Slothe is close to 1 (i.e., most individuals behave similar to the median), while for desynchronized populations the Sloexp/Slothe ratio is smaller (i.e., many individuals deviate from the median). C) In addition, we calculated the percentage of flies for each genotype with a Sloexp > ½ of the Slothe where a high versus low percentage again indicates synchronized or desynchronized behavior, respectively. Indeed, > 80% of the control flies increase their locomotion with Sloexp > ½ Slothe, compared to only 50–60% of the Mai179>cycDN and cry[19]>cycDN flies (S1C Fig, blue bars percentage of flies with Sloexp > ½ of Slothe ‘anticipating’, orange bars percentage of flies with Sloexp < ½ of Slothe ‘non-anticipating’). Same flies as in Fig 1B–1E). (TIF) [file pgen.1010487.s001.tif]

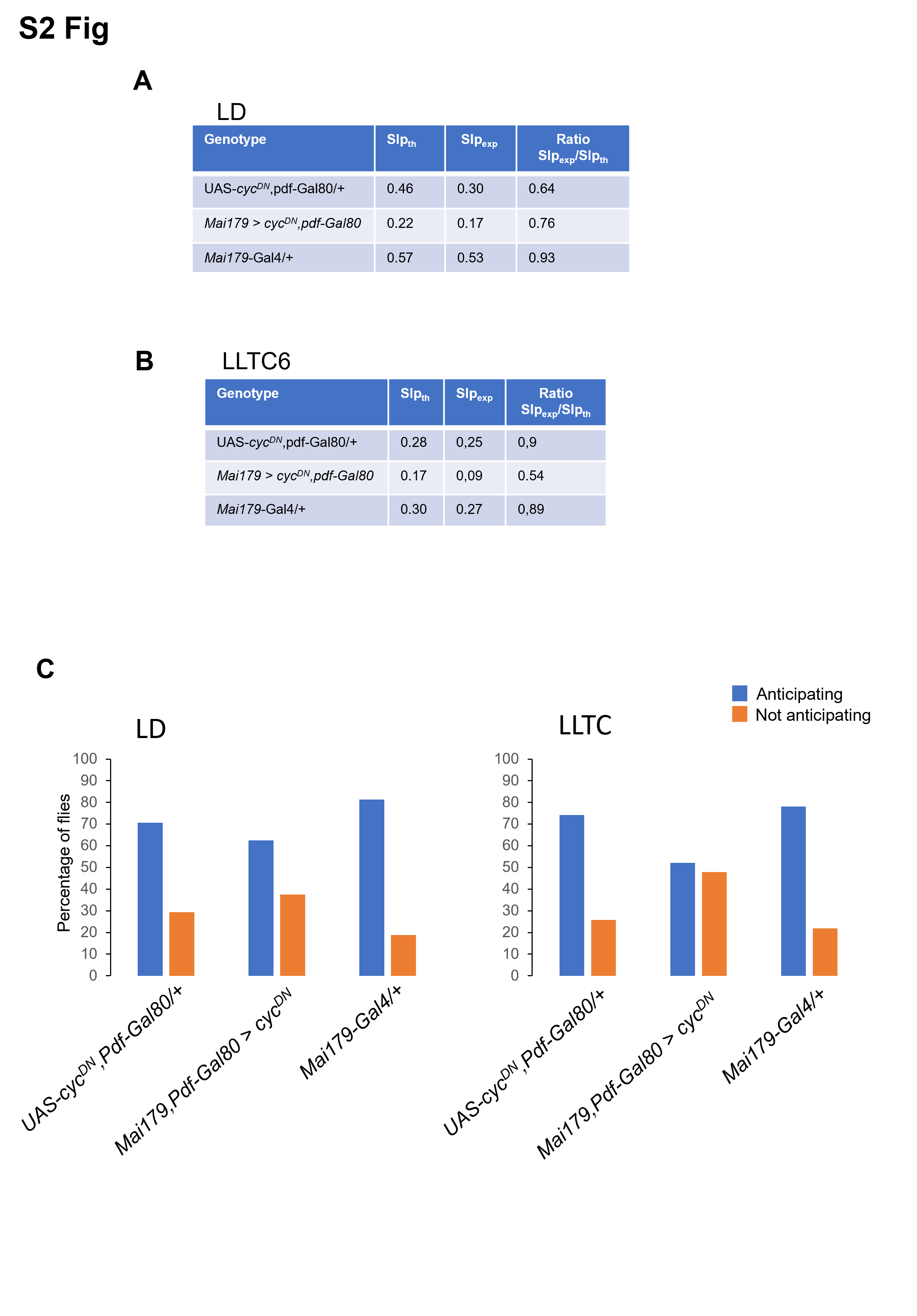

Supplement: S2 Fig — A-B) Slothe, Sloexp values and the ratio Sloexp/Slothe of the indicated genotypes in LD (A) and on day six of LLTC (B). C) Percentage of flies anticipating (blue) and not anticipating (orange) lights-off (left) or the temperature decrease (right), defined as described in the legend of S1 Fig and in Materials and Methods. Same flies as in Fig 3C–3D. (TIF) [file pgen.1010487.s002.tif]

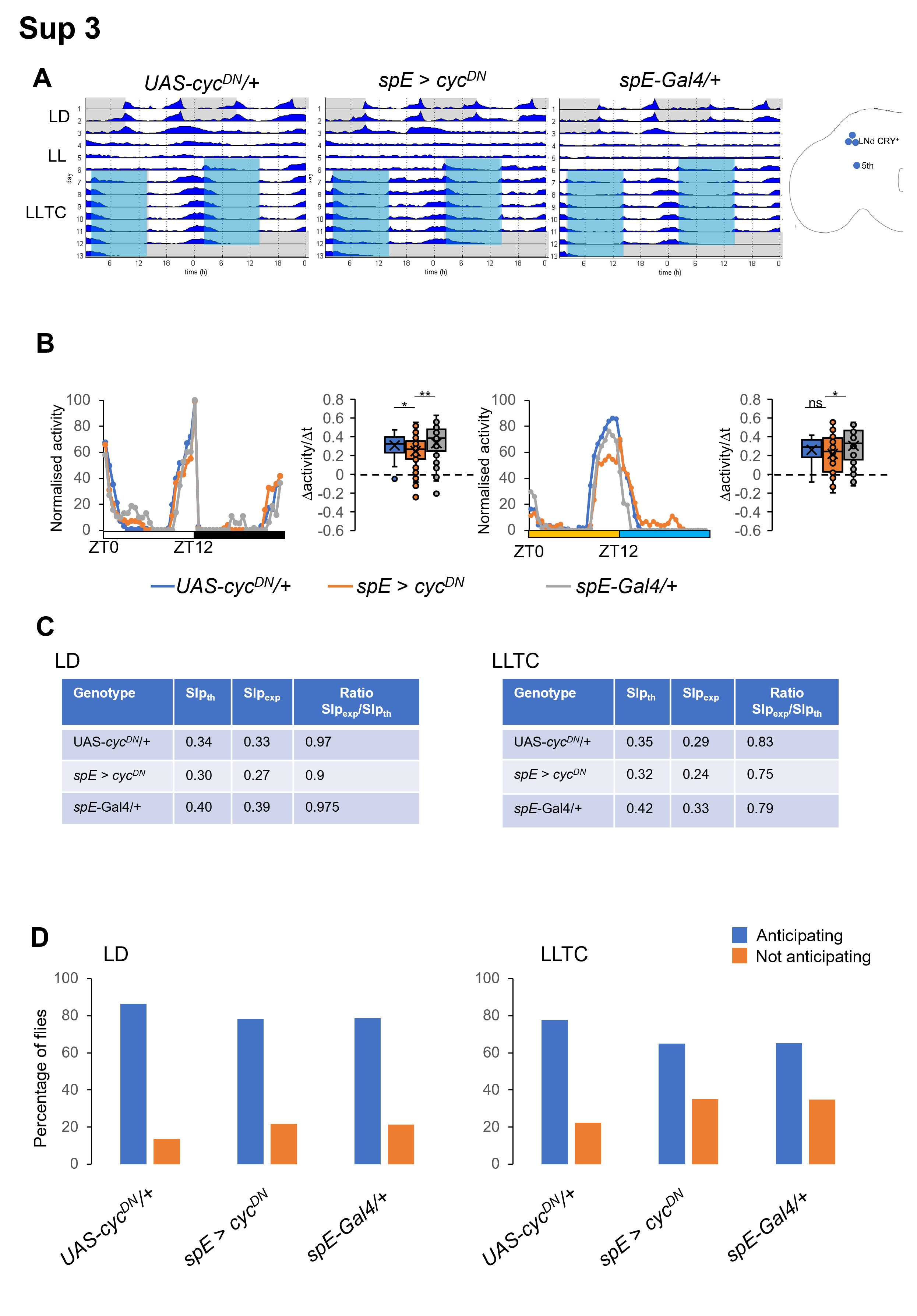

Supplement: S3 Fig — A) Double plotted average actograms of the indicated genotypes. Cartoon on the right shows the clock neurons expressing spE-Gal4. N: UAS-cycDN/+ = 19, spE > cycDN = 20, spE-Gal-4/+ = 19. B) Median of normalized locomotor activity (left) and slope (right) in LD and LLTC6. N: UAS-cycDN/+ = 58, spE > cycDN = 60, spE-Gal-4/+ = 46. Statistical test: Kruskal wallis [57]. *: p<0.05, **:p<0.005, ***:p<0.001. C) Values of the Slothe, Sloexp and the ratio Sloexp/Slothe of the indicated genotypes in LD and LLTC6. Same flies as in B. D) Percentage of flies anticipating (blue) and not anticipating (orange) lights-off (left) or the temperature decrease (right), defined as described in the legend of S1 Fig and in Materials and Methods. Same flies as in B. (TIF) [file pgen.1010487.s003.tif]

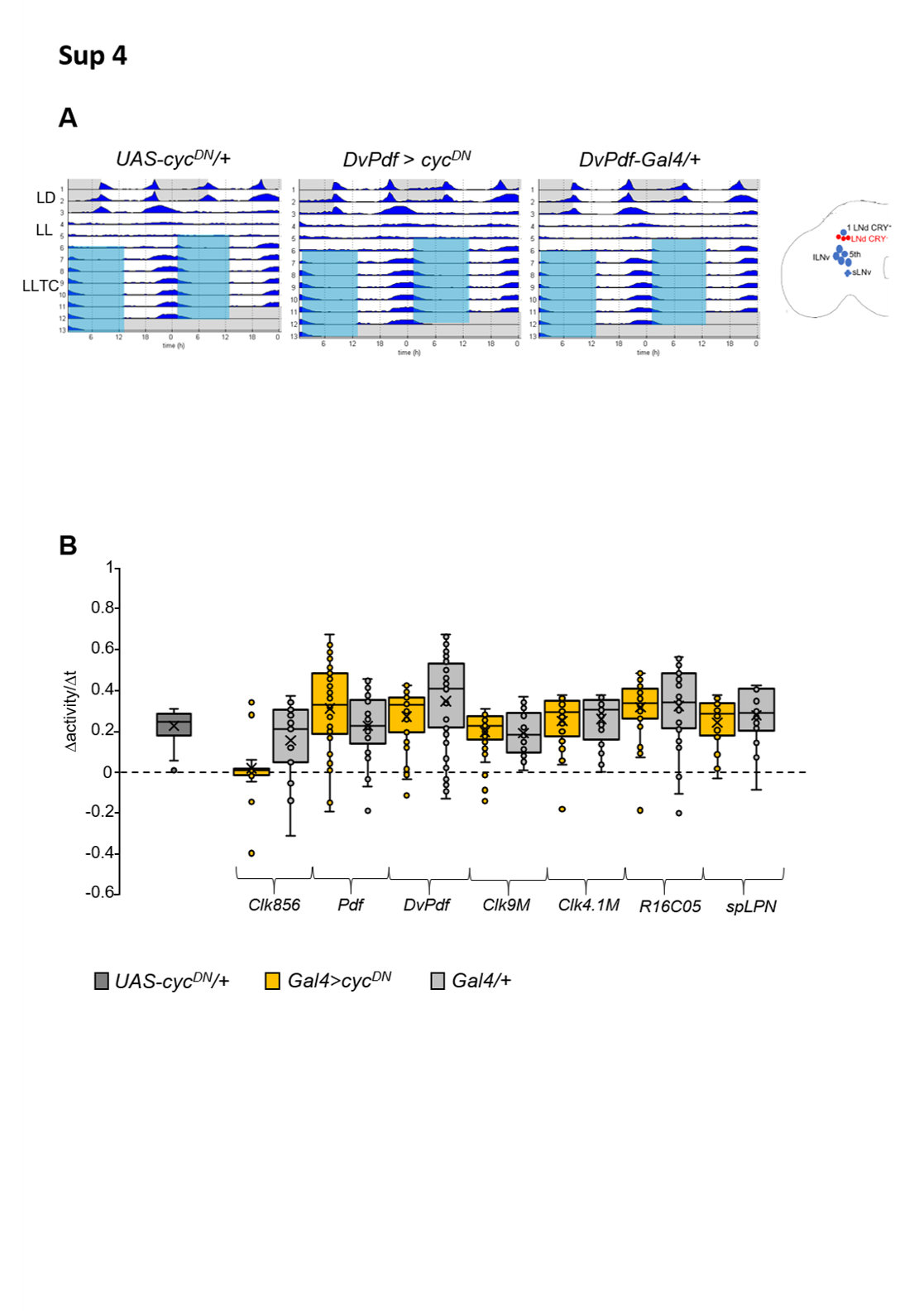

Supplement: S4 Fig — A) Double plotted average actograms of the indicated genotypes. Cartoon on the right shows the clock neurons expressing DvPdf-Gal4. Note the LNd CRY- are in red. N: UAS-cycDN/+ = 18, DvPdf > cycDN = 20, DvPdf-Gal-4/+ = 19. B) Box plots showing the slope of the evening peak on the 6th day of LLTC. N: UAS-cycDN/+ = 172, 20 ≤ Gal4>cycDN ≤ 41, 18 ≤ Gal4/+ ≤ 40. (TIF) [file pgen.1010487.s004.tif]

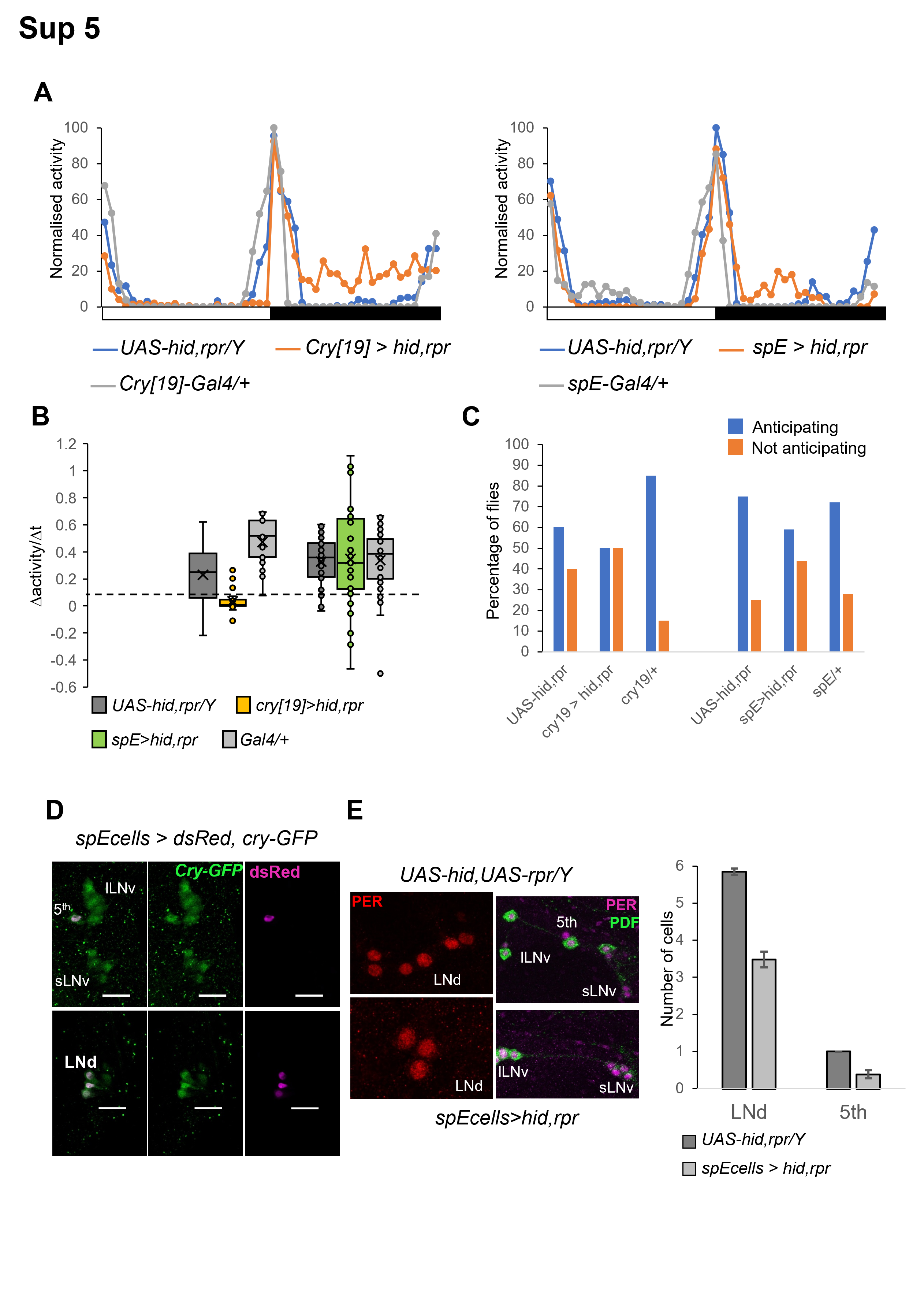

Supplement: S5 Fig — A) Median of normalized locomotor activity in LD. Same flies as in Fig 4B. B) Box plots showing the slope of the evening peak in LD. Same flies as in Fig 4B and 4C) Percentage of flies anticipating (blue) and not anticipating (orange) lights-off in LD. D) Immunostaining of lateral neurons. spE-Gal4 drives expression of the nuclear marker dsRed, CRY+ neurons are labeled by expression of a GFP-CRY fusion protein [53]. Scale bar 10μm E) Immunostaining and quantification to determine the number of ablated neurons after hid and rpr expression in spE cells. Flies were dissected at ZT2 in LD. The graph on the right shows the average number of LNd and 5th-sLNv cells observed. Number of brains per genotype is 11. (TIF) [file pgen.1010487.s005.tif]

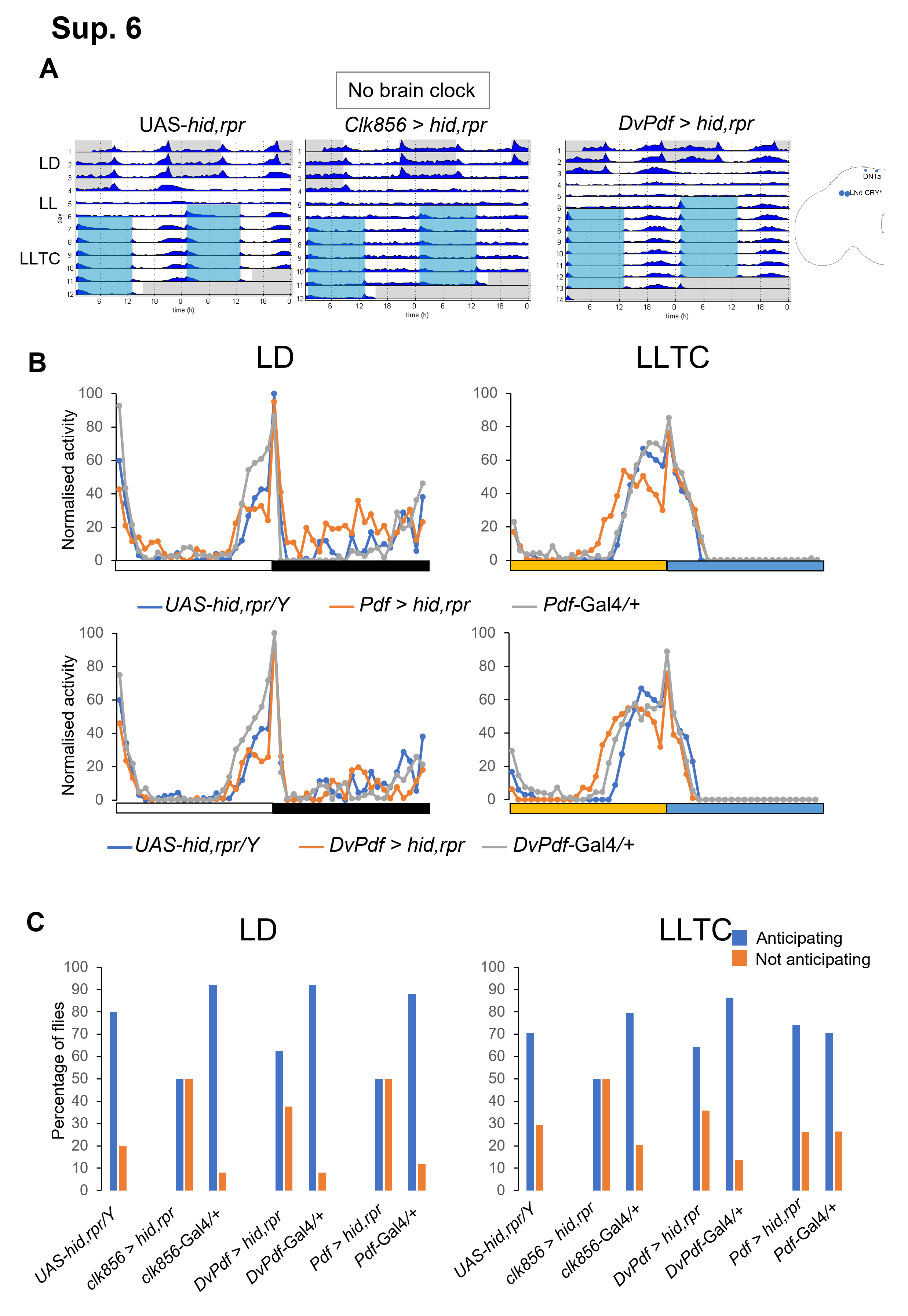

Supplement: S6 Fig — A) Double plotted average actograms of the indicated genotypes. Cartoon on the right showing morning and evening clock neurons, remaining after ablation using the DvPdf driver. N: UAS-hid,rpr/Y;ls-tim = 21, Clk856>hid,rpr = 24, DvPdf>hid,rpr = 24. B) Median of normalized locomotor activity in LD (left) and during day six of LLTC (right). N: UAS-hid,rpr/Y;ls-tim = 34, Pdf > hid,rpr = 50, Pdf-Gal4/+ = 34, DvPdf>hid,rpr = 28, DvPdf-Gal4/+ = 44. C) Percentage of flies anticipating (blue) and not anticipating (orange) lights-off in LD (left) or the temperature decrease (right). Same flies as in B, and N for Clk856>hid,rpr = 24, clk856-Gal4/+ = 44. (TIF) [file pgen.1010487.s006.tif]

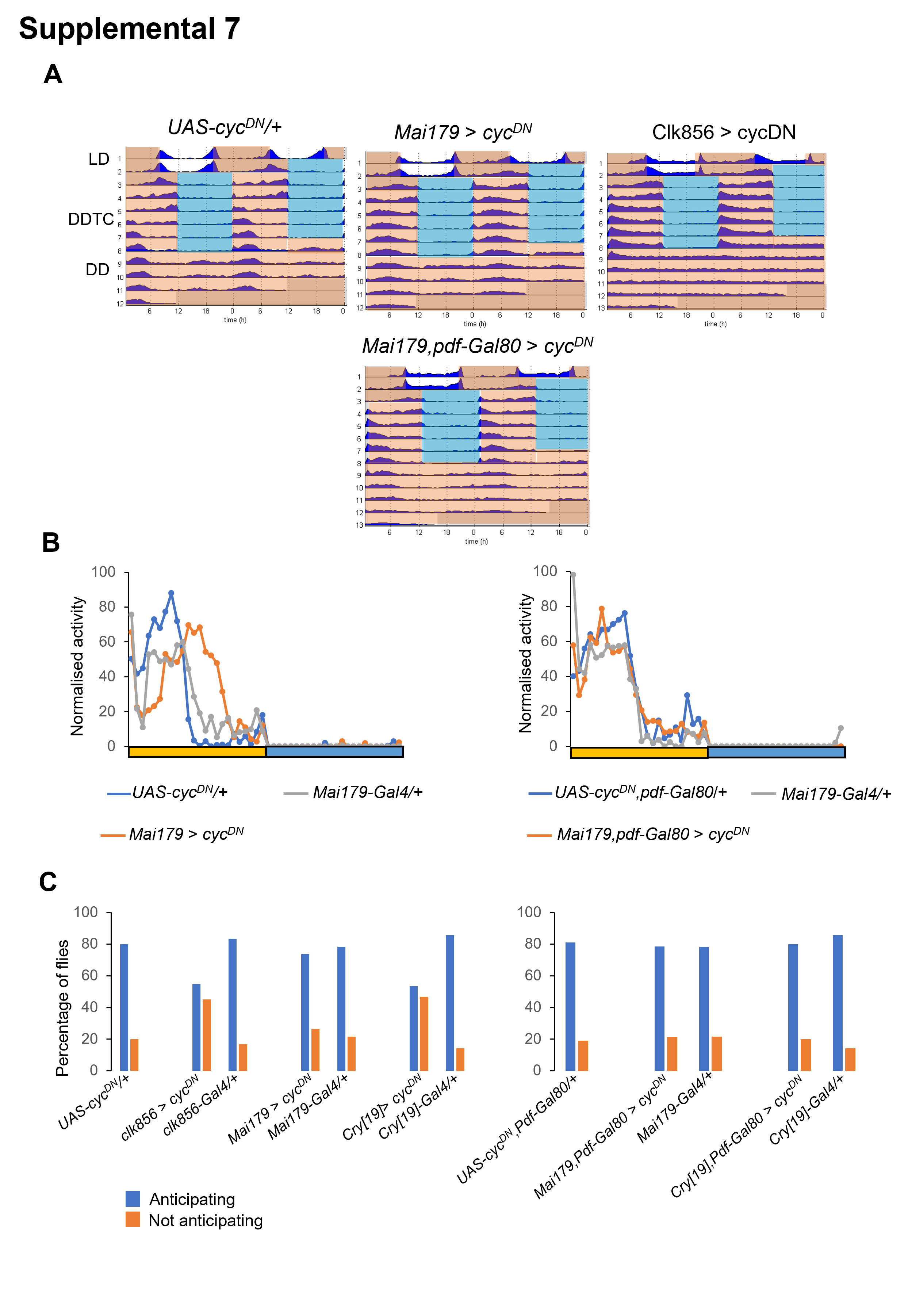

Supplement: S7 Fig — Double plotted average actograms of the indicated genotypes. N: UAS-cycDN/+ = 20, Mai179>cycDN = 19, Clk856>cycDN = 31, Mai179,pdf-Gal80>cycDN = 14. B) Median of normalized locomotor activity during the 6th day of DDTC. N: UAS-cycDN/+ = 20, Mai179>cycDN = 19, Mai179-Gal4/+ (left) = 20, UAS-cycDN,pdf-Gal80/+ = 21, Mai179,pdf-Gal80>cycDN = 14, Mai179-Gal4/+ (right) = 17. C) Percentage of flies decreasing their locomotion with a slope steeper (blue), or lower (orange) than 50% of the theoretical slope after the morning peak at DD2 following DDTC. Same flies as in panel B and Fig 5C. (TIF) [file pgen.1010487.s007.tif]
