# Supplementary material for: Light triggers a network switch between circadian morning and evening oscillators controlling behaviour during daily temperature cycles
Source: PLoS Genet. 2022 Nov 11;18(11):e1010487. doi: 10.1371/journal.pgen.1010487 (PMC9683589; doi:10.1371/journal.pgen.1010487)
Supplement: Attachment — Submitted filename: Response to Reviewers.docx [file pgen.1010487.s009.docx]

**All our answers to the reviewers below are in *italics***

Reviewer #1: Review uploaded

Using the features of locomotor activity in *Drosophila melanogaster* Loerber et al., propose the idea that components of the circadian pacemaker circuit have clear roles in determining activity at a certain phase regardless of the zeitgeber. Experiments under constant light and cycling temperature have been conducted to support the classification of a group of neurons in the fly circadian circuit as ‘Evening cells’.

The claims made in this manuscript can be considered partially novel.

- Some of the dorsal neurons are known to be entrained by temperature cycles. Picot et al showed that even in the larval stage if light input or input from the lateral neurons to the dorsal neurons is absent, the clock in dorsal neurons can be entrained by temperature cycles and they can also set the phase of the neurons in the circuit under these conditions.

Picot, M., Klarsfeld, A., Chelot, E., Malpel, S., & Rouyer, F. (2009). A Role for Blind DN2 Clock Neurons in Temperature Entrainment of the Drosophila Larval Brain. In Journal of Neuroscience (Vol. 29, Issue 26, pp. 8312–8320). Society for Neuroscience. <https://doi.org/10.1523/jneurosci.0279-08.2009>

- Busza et al has characterised the circadian circuit and the role of M and E cells under constant darkness with temperature cycles (DDTC) in adult fly. It demonstrated that while Evening neurons show high responsiveness to temperature, Morning neurons may resist behavioural synchronization to temperature cycles. Altogether, the properties of each neuronal group and their coupling would thus, regulate the phase of activity under DDTC.

Busza, A., Murad, A., & Emery, P. (2007). Interactions between Circadian Neurons Control Temperature Synchronization of Drosophila Behavior. In Journal of Neuroscience (Vol. 27, Issue 40, pp. 10722–10733). Society for Neuroscience. <https://doi.org/10.1523/jneurosci.2479-07.2007>

- Miyasako et al tracked the molecular clock component of TIMELESS in the circadian neurons in the adult fly brain under conditions with both light and temperature cycling. The temperature cycle was advanced by 6 hours compared to the light cycle. Using this they demonstrated that while lateral neurons are primarily light entrainable, the dorsal and lateral posterior neurons preferentially entrain to temperature cycle.

Miyasako, Y., Umezaki, Y., & Tomioka, K. (2007). Separate Sets of Cerebral Clock Neurons Are Responsible for Light and Temperature Entrainment of Drosophila Circadian Locomotor Rhythms. In Journal of Biological Rhythms (Vol. 22, Issue 2, pp. 115–126). SAGE Publications. <https://doi.org/10.1177/0748730407299344>

The current study can thus, support the role of E cells in synchronization of activity to temperature cycles under constant light as well.

Information from the previous literature has not been discussed to provide a background for the hypotheses framed nor have the results been put into perspective and context of previous literature. Three such studies have been mentioned above.

*We agree with the referee that previous studies on the role of various clock neuronal groups in temperature entrainment have been performed. However, the underlying questions of these studies were different from the current, and no previous study focussed on the role of evening oscillator neurons in temperature entrainment during constant light. As we showed in a recent publication, temperature synchronisation in constant light is of great importance from an ecological and evolutionary perspective (Lamaze et al 2022, Nat Commun 13:1724 doi:* *10.1038/s41467-022-29293-6): at least some species living in Northern latitudes (i.e. long summer days) appear to rely on the ability to synchronize their clocks to temperature cycles in order to elicit synchronized behaviour. Nevertheless, in the revised version of the manuscript we made an effort to put our findings in the context of previous publications, in particular those reported by Busza et al (2007) and Gentile et al (2013), which use similar approaches and methodology as we applied here.*

The observations reported here do support their claims to some extent.

The term synchronization has not been explained or defined the beginning and thus, it is not immediately clear what is the phenotype or activity pattern is being scrutinized for differences. The details of the measure used to quantify the differences in phenotype (slope calculation) has been explained, however, the rationale for using this measure seems unclear.

*We thank the reviewer for pointing this out and we now clearly define ‘synchronisation’. We give a more detailed and clearer explanation of the slope calculation. The steepness of the slope (Δactivity/Δt*) *describes the speed of anticipatory behavioural activity increase before the temperature transition in the evening. We calculate slope for each individual on the 6^th^ day of LLTC, which gives us a measure of the amplitude of anticipatory behaviour. These slope values are plotted as box plots throughout the manuscript. In order to define, and at the same time quantify synchronization, we now compare the slope of each individual to the theoretical slope calculated based on the median of the population. The idea here is that if the individuals of a population are synchronized to each other, most individual slope values (=slope_experimental)_ will be similar to the median (slope_theoretical_), resulting in a ratio of median experimental to median theoretical slope close to 1. If the population is less well, or not synchronized, this ratio will be clearly below 1. These ratios are tabulated in the revised version of the manuscript or specified within the results text. Finally, we calculated the percentage of flies for each genotype with an experimental slope higher than half the value of the theoretical slope where a high versus low percentage again indicates synchronized or desynchronized behavior, respectively. These percentages are also plotted with the respective figures of the revised manuscript.*

**Analyses**

1) The activity profiles are only of one particular day under the regime averaged across individuals. The error across individuals has not been plotted in the profile. Typically activity profiles are averaged across days (post stable entrainment, if the regime is changes) and then across individuals. In this manuscript, in the average profiles which are used to compare across genotypes, information across cycles under a particular regime is lost.

*The reviewer is correct in that we performed the formal analysis and behavioural quantification described above on the last day of temperature entrainment (day 6 of LLTC). Day 6 is chosen, because by then all the flies should have been synchronized to TC (or not) even if synchronisation may take more time in certain genotypes. In other words, with our method we can exclude that potential transient behaviour during TC interferes with the results and their interpretation. In addition, this method allows for easy combination of several experiments, further increasing the informative value of our results. Moreover, in order to focus on the activity profile, we decided to plot the median of the normalised activity. The median is a better representation compared to the mean, because the latter relies on normally distributed data, which is not always the case (in particular when studying small population sizes). For these reasons, this kind of analysing locomotor activity data (i.e. analysing data from one 24-period from many individuals and across experimental repeats) is frequently used in the sleep field (e.g., Hendricks et al 2000 Neuron, 25: 129, Shaw et al 2000 Science, 287:1834).*

*Nevertheless, we do supply average actograms of fly populations showing average activity on each single day during the entire experiment (LD, LL, and LLTC), to allow the reader judging the actual behaviour for the potential presence of transient behaviour during certain parts of the experiment, and to visualize activity peaks (or the lack thereof) as well as anticipatory behaviour*. *We feel that in this way we supply a very comprehensive display and analysis of behavioural activity.* *However, if the reviewer requests, we will also supply averages across multiple days for defined periods of the entire experiment.*

2) The PER quantified using IHC in the circadian neurons has not been statistically tested for significant circadian oscillation (for example by using repeated measures ANOVA).

*We thought it was very clear if the various neuronal groups showed oscillations or not (we did include the SEM for each time point as well). Nevertheless, for the revised version of the manuscript we performed statistical tests to demonstrate significance of the circadian oscillations or the lack thereof.*

There are certain inconsistencies in the driver lines used. For example in drivers specific to M cells (*pdfgal4*) and for E cells (*spEgal4*) have not been used throughout under LLTC regime systematically to delineate the role of these cells groups and under DDTC (in the final result section).

*We agree with the referee. Part of the reason for the use of different drivers is that the spE driver, although very specific, is weaker compared to other evening drivers we uses (Mai-179 Mai179/Pdf-gal80. We demonstrate this difference in driver strength in the revised version of the manuscript (Fig2 and 3 for Mai179 and Mai179/Pdf-gal80, Figure S5 for spE-gal4). As suggested by the reviewer, we also now supply data with all drivers for expressing cycDN, as well as for the DDTC experiment. For the ablation experiments this was not possible because of lethality associated with the Mai179 driver expressing UAS-hid,rpr. The results show in general weaker genotypes with the spE driver which can be explained by the weaker expression of this driver. The results also depend on the specific manipulation, as for example expression of cycDN requires strong expression to achieve dominance over endogenous wild type CYC, while hid, rpr expression even at a low level is expected to induce apoptosis. Indeed, with the spE driver we observe a stronger phenotype with UAS-hid, rpr ablation compared to UAScycDN expression. Combining the results of all drivers and manipulations, the experiments clearly confirm the role of the evening neurons for driving evening behaviour in LLTC and a switch to the morning neurons in DDTC.*

Using the above mentioned specific drivers and assaying their activity and PER profiles under LLTC can make the results clearer and more robust. The manipulation using these drivers using cell ablation for some results and expression of a dominant negative mutant to dysfunction the clock in most others. Ablation studies are generally considered drastic especially since the advent of other mutants which can be used to manipulate the speed of the clock (and can thus help study the influence of clock in specific cell groups on the timing activity bouts).

*As outlined above, we now supply data for most drivers for each of the behavioural assays. We think that adding experiments involving changing of clock speed could actually result in confusion. Changing the speed of the clock in specific groups of neurons helps a lot to understand the circadian network hierarchy under constant conditions, but doing that during alternating environmental conditions we would be adding up multiple parameters which would complicate, or even prevent proper interpretation and conclusion.*

Result-wise comments

1) The evening cells control the evening activity during temperature cycles in constant light.

Typos in line 6 and 11 of the first paragraph.

*Typos have been corrected*

The statement ‘As expected, expression of *cyc^DN^* in the morning and evening oscillator with both drivers, strongly reduced the amplitude of the evening peak in LD’ does not seem fully accurate because the evening peak resulting from the startle is just as high in experimental flies compared to controls. The overall evening activity bout does however, seem dampened. Under LLTC, for experimental flies there also activity visible in the cryophase (Fig.1C)

*We apologize for not making ourselves clearer. We refer here to the anticipatory behaviour leading up the sudden change of the environmental condition (lights off in LD; temperature drop in LLTC). The reviewer is correct, that the actual startle activity response to lights off in LD is not altered in the experimental cycDN flies. While the startle response is not controlled by the circadian clock, the anticipatory behaviour (in this case the evening peak) is, and it is the amplitude of this clock controlled evening peak, which is reduced by cycDN expression in clock neurons. The referee is correct that this manipulation also results in increased activity during the cryophase of LLTC. We now mention this in the results part, but since we focus on the evening anticipatory peak, we did not further study this increased activity. We changed the relevant section in the results part to the following:*

*‘*As expected, expression of *cyc^DN^* in the morning and evening oscillator with both drivers strongly reduced the amplitude of the evening peak, which anticipate the environmental transition in both LD and LLTC (Fig 1A-C). While the startle response to lights-off and the following rapid activity decline in LD was not affected, both *Mai179-Gal4* and *cry-Gal4[19]* > *cyc^DN^* flies showed increased activity in the cryophase during LLTC (Fig 1 A-C) In the following we focus on the amplitude of the anticipatory behavioral evening peak, because it is under tight clock neuronal control and also inferenced by the environment.’

Paragraph 2 ‘In particular, neither *Pdf-Gal4* nor *DvPdf-Gal4* which are both expressed in the morning cells but not in the evening cells, had an effect on the evening peak’. *DvPdf-Gal4* is known to be expressed in at least two of the evening cells (5^th^ sLNv and one CRY^+ve^ LNd). Thus, the statement is not accurate. It is possible that the differences in the extent to which all the E cell group is targeted by these and other E cell specific drivers results in the differences in activity patterns. However, this will need to be discussed and elaborated upon.

*The reviewer is correct, but we did say that DvPdf is expressed in 2 of the 6 evening neurons (see also Table 1). The point here was to show that expression of cycDN in ALL PDF+ neurons does not interfere with evening peak synchronization in LLTC. To make this clearer, we changed the respective results section in the following way:*

‘In particular, neither *Pdf-Gal4* nor *DvPdf-Gal4* which are both expressed in all of the PDF^+^ morning cells, had an effect on the evening peak (S4 Fig). In agreement with previous findings (19), this suggests that a functional clock in the morning cells is not necessary for synchronizing behavior in LLTC. Moreover, the *DvPdf-Gal4* result shows that expression of *cyc^DN^* in only two out of the six evening neurons (1 CRY^+^ LNd and the 5^th^ s-LNv, Table 1) is not sufficient to interfere with synchronizing evening activity in LLTC (S4 Fig).’

2) Morning and evening oscillator neurons are not required for temperature entrainment of other clock neurons

Few other studies have shown that dorsal neurons can be entrained by temperature cycles even in the absence of M and E cells. The current results support a similar claim under a different regime. The result has not been discussed in the context of these previous findings.

*The reviewer is correct, and we now cite and discuss these papers (Yoshii et al 2005, Busza et al 2007) in the relevant section of the Discussion. The revised text reads as follows:*

‘Nevertheless, even in the absence of all CRY^+^ neurons, flies exhibit weak synchronized behavior with an evening phase during temperature cycles (in LL and DD), suggesting that CRY^-^ clock neurons (subsets of DN1_p_ and LNd, DN2, and DN3) also play a role in temperature synchronization (24,31). Similarly, ablation of PDF^+^  or all three groups of LN (LNv, LNd, LPN) still allows for weak behavioral evening synchronization to 25°C : 30°C TC in LL (19). Furthermore, the fact that in the absence of a clock in the Mai179^+^ cells (Fig 2), after ablation of all CRY^+^ cells (31), or in the absence of all LN (19), molecular oscillations in other clock neurons can be synchronized, supports the idea that multiple and independent pathways contribute to temperature entrainment (19,27,29–31,40).’

3) The evening clock neurons are necessary for synchronized behavior in LLTC but not in LD

It has been studied and long known in the field that E cells affect E activity bout. (Although in the previous studies *cyc^DN^* was not used.) The classification of E cells was on the basis of regulating of evening bout of activity under LD 12:12. This result might weaken the novelty of the claim of deterministic role of E cells under different zeitgeber conditions.

*We are not sure to understand the point of the reviewer here. What we are proposing is that a group of clock neurons determines specific timing of behavioural activity under a particular environmental condition regardless of the zeitgeber being used (light or temperature). In both LD and LLTC the CRY+ evening cells control the timing of the circadian locomotor output to occur in light and 25°C conditions. In contrast, current models propose that CRY positive neurons mediate behaviour under light:dark conditions, while the CRY negative neurons control behaviour under temperature cycles (e.g., Yoshii et al 2010. J Biol Rhythms). Here, we demonstrate that the shape of the rhythmic behaviour does not depend on the zeitgeber used for entrainment (LD or LLTC). Hence, in DDTC another group of clock neurons (the morning cells) takes over the control of the rhythmic behaviour. We have made this point clearer in the Discussion of the revised manuscript.*

5) Role of the evening cells under DDTC

The background or rationale to the hypothesis in this section is not clear. The study, Busza et al., 2007 provides a thorough characterisation of the role of M and E cells under temperature cycles in DD. The current results will have to be discussed in its context.

*The referee is correct that role of M and E cells has been analysed in DD and temperature cycles (Gentile et al 2013, Busza et al 2007). The point we wanted to make here is that in DDTC the evening neurons are not needed for synchronization. We expressed cycDN in these neurons (now using cry-gal4/Pdf-gal80 in addition to Mai179 to increase specificity), and observed synchronized behaviour with a morning phase like in wild type flies, suggesting that the morning neurons now mediate synchronization. These results nicely fit with previous studies showing that PER rescue in morning neurons only, also results in synchronized morning activity in DDTC (Gentile et al, Busza et al). As the reviewer requested, we refer to the results of these studies at the relevant section of Results, and in the Discussion of the revised version of the manuscript.*

**General comments regarding the figures**

For each figure a cartoon might be helpful to show which cells/cell groups (for example M versus E) have a functional clock or are being targeted for ablation.

*We now indicate in each Figure which neurons were manipulated by a cartoon.*

What are the error bars in activity profiles?

*We think the reviewer refers to the box plots? There are no error bars in the activity plots, because we show the median, not the average. We now give a detailed description of the whiskers and lines depicted in the box plots in the legend to Figure 1.*

Demarcation of thermophase and cryophase in actograms – Post transfer to LLTC the grey shade is used for cryophase (16 ^o^C). However, under LD grey shade is 25 ^o^C darkness. It would be better to make the shading clearer. In Fig 5 the advance of thermocycles with respect to the LD cycle before is not clear in the actograms.

*Although we indicate the conditions during each experiment to the left of the actograms, we agree with the reviewer. To make the actograms clearer we now coloured the cryophase in LL and DD in blue.*

Reviewer #2: In this manuscript Lorber and colleagues examine the role of the so-called evening oscillators in the control of daily locomotor rhythms under temperature cycles.  The question of how the clock neuron network operates under distinct environmental conditions is a critical one, as clocks achieve their adaptive value by orchestrating daily rhythms so that behavioral and physiological outputs occur at the proper times relative to predictable environmental changes.  Thus, the central question posed here is of great interest to the field of chronobiology.

The authors show that the evening cells largely maintain their role as promotors of late-day activity, even under temperature cycles under constant light and argue that this suggests “a more deterministic function” of clock neuron sub-classes.  Though the central theme is of significant interest and there is admirable technical rigor in most experiments, conceptual and methodological concerns diminish enthusiasm for the study as currently presented.

Major Concerns:

The central conclusion of “a more deterministic function” for E oscillators leads to the question “more deterministic than what?”  The authors have not described a coherent alternative model to the longstanding two oscillator model that might lead to the prediction of a “less deterministic function” for these cells.  Without a clear rational for a “less deterministic” mechanisms of behavioral control than the currently widely shared model, the novelty/impact of the present work is not at all clear.  Was there a compelling reason to think that the established evening oscillators would not govern late day activity under most 24h environmental cycles in which a “day” could be defined?  Was there a reason why we would expect a different role for evening oscillators under temperature cycles and constant light?

*We apologize for the confusion we caused by using the term ‘deterministic’. What we meant to say is simply that the evening neurons control behavioural activity both under LD and LLTC conditions. We do not think that this could have been expected a priory, because previous work proposed that CRY+ neurons mediate synchronization to light.dark cycles, while CRY- clock neurons are more important for temperature entrainment (see response to reviewer 1 and Yoshii et al 2010, for example). Moreover, we and others have shown that in DDTC CRY+ morning neurons synchronize behavioural activity to occur in the morning (Figure 6, S7, Gentile et al 2013, Busza et al 2013). In the revised version of the manuscript we omit the term ‘deterministic’ and instead clearly state the role of the CRY+ evening neurons for synchronisation in LD and LLTC, and the switch to the morning neurons in DDTC.*

The authors have done an admirable job in their attempts to confirm that genetic manipulations worked as designed.  If only everyone were this careful.  However, the effects of Dominant-Negative-clock mosaicism appeared to be quite variable, particularly for the critical Mai-179-GAL4/Pdf-GAL80 mosaics.  Furthermore, the ablation experiments using the split Evening oscillator driver were not uniformly effective, often sparing neurons from ablation.  The authors dismiss this as an explanation for the absence of a behavioral phenotypes under LD because spared cells were never seen in both brain hemispheres, but there is precedent for one hemisphere being enough to support timekeeping function: a single s-LNv in one hemisphere was likely sufficient for the maintenance of free running locomotor rhythms (Helfrich-Förster 1998 J. Comp. Physiol. A V.182, p.435).  Thus, the presence of spared E cells seems like a viable explanation for the lack of behavioral phenotypes under LD, and make the interpretation of (rather subtle) effects in LLTC difficult to interpret.

*The referee is of course correct, and we now discuss the lower penetrance of the spE-Gal4 driver (demonstrated in Fig S5) as the most likely explanation for the absence of a behavioural phenotype in LD and LLTC in combination with cycDN:*

‘Interestingly, while restoring PER expression in these neurons is sufficient to restore evening anticipation in LD (S9 Fig), expression of *cyc^DN^* does not alter the shape of behavioral evening activity in both LD and LLTC (S3 Fig). Although this may indicate a prominent role for the DN1a in regulating evening activity, we think the weak expression of the *spE-Gal4* driver allows for sufficient clock function in the 5^th^ s-LNv and the CRY^+^ LNd evening cells of *spE > cyc^DN^* flies (see S5 Fig).’

Given the focus here on evening oscillators, it is surprising to see no effort to use Pdf-GaL80 along with Mai-179-GAL4 for the experiments assessing rhythms under DDTC.  The authors state that this driver is expressed at lower levels in the morning cells than in the evening oscillators, but we would still expect the expression of dominant negative cycle to have an effect on Morning Oscillator timekeeping.  It is also troubling not to see the split Evening cell driver used here.  Why did the authors not apply the same tools to DDTC that they did to LLTC?

*The reviewer is right. We now crossed the recombinant containing Pdf-gal80 and UAS-cycDN on the same chromosome to Mai179-gal4 and cry19-gal4, to specifically express cycDN in the CRY+ evening neurons. The results confirm the results obtained with Mai179 driver without Pdf-gal80 and show that flies without a functional clock in E cells, but with a functional clock in M cells, synchronize their activity in DDTC with an activity phase in the morning (new Figure 6 and S7 in the revised manuscript). Together with previous studies (Busza et al 2007, Gentile et al 2013) these findings indicate that PDF+ morning neurons synchronize flies to DDTC with a morning phase. We discuss our findings in the context of the previous publications in the revised version of the manuscript.*

Though the authors discuss their results in the context of recent work on the fly clock neuron network, they do not present a coherent alternative model of clock network function based on these new results, nor is there a clear or compelling revision in our conception of the network or how it entrains to distinct environmental rhythms.

*We did not intend to demonstrate how the brain clock is entrained by temperature cycles. We are focusing on how clock neurons shape the behaviour under specific environmental conditions regardless of the Zeitgeber. In the revised version we no discuss our findings in the context of what was known from previous papers that study temperature synchronization in DDTC and LLTC. The two main points we found and discussed are: 1) Contrary to expectations based on previous studies CRY+ evening neurons (as opposed to CRY-negative clock neurons) are important mediators of temperature synchronization in LL. 2) blocking the function of these evening cells has no effect on synchronisation to DDTC, in line with previous studies implicating PDF+ morning neurons for clock synchronization under these conditions, and nicely demonstrating a neuronal network switch going from LLTC to DDTC.*

Minor Concerns:

Page 4, Line 8: What do the authors mean by “restrictive light conditions?”

*What we meant was ‘low light’, but the relevant passage has been deleted from the revised manuscript.*

As written the authors suggest the Driver Dv-Pdf-GAL4 “is expressed in the morning but not evening cells,” (p. 6, line 11), but this is inaccurate. This driver is also expressed in subsets of Evening oscillators (Bahn et al. 2009 Genetics V.181, p. 965; Guo et al. 2014 ELife V.3:e02780).

*See above our response to reviewer 1. The reviewer is correct, but we did say that DvPdf is expressed in 2 of the 6 evening neurons (Table 1) and we now also clearly write this in the results text. The point here was to show that expression of cycDN in ALL PDF+ neurons does not interfere with evening peak synchronization in LLTC. To make this clearer, we changed the respective results section in the following way:*

‘In particular, neither *Pdf-Gal4* nor *DvPdf-Gal4* which are both expressed in all of the PDF^+^ morning cells, had an effect on the evening peak (S4 Fig). In agreement with previous findings (19), this suggests that a functional clock in the morning cells is not necessary for synchronizing behavior in LLTC. Moreover, the *DvPdf-Gal4* result shows that expression of *cyc^DN^* in only two out of the six evening neurons (1 CRY^+^ LNd and the 5^th^ s-LNv, Table 1) is not sufficient to interfere with synchronizing evening activity in LLTC (S4 Fig).’

The authors state that they were unable to make use of the Pdf-GAL80 element in their Cry-GAL4 ablation experiments “for genetic reasons.”  The reasons here are not immediately obvious and this would have been a very informative experiment.  The authors should elaborate on why this combination was not possible.

*As outlined above, we now successfully generated a recombinant containing Pdf-gal80 and UAS-cycDN on the same chromosome, which allowed us to specifically express cycDN in the CRY+ evening neurons (cry19-Gal4 > cycDN, Pdf-Gal80* and *Mai179-Gal4 > cycDN, Pdf-Gal80). The results obtained with this line are shown in Figure 6 of the revised manuscript.*

Page 9, second paragraph of discussion section: the author state that s-LNv output is inhibited by light.  Though they cite a study that inferred this from a series of genetic manipulations under different conditions, it is difficult to accept this as settled fact in the face of compelling and direct evidence that the morning cells are acutely and robustly excited by light input (Schlichting 2016 J. Neurosci. V.36, p. 9084; Li et al. 2018 Nat. Commun. V.9, p. 4247).

*We agree with the reviewer that there are several reports indicating that morning cells are directly and indirectly activated by light. However, Cusumano et al clearly demonstrate that in presence of light and when the clock of the sLNv is functional flies are arrhythmic. Yet, after removal of the visual system (GMR-hid) flies become rhythmic in the presence of light, indicating at least an inhibiting effect of the visual system on s-LNv output. Nevertheless, in order to avoid unnecessary complexity, we decided to remove the relevant sentence and the following one from the Discussion*

The authors use the word “dampening” to describe lower amplitude evening peaks under entrained conditions throughout the text.  Given that this term has a very different and commonly-understood meaning in the context of rhythms, i.e., a prolonged and gradual reduction in the amplitude of free-running rhythms, the authors should consider revising.

*We agree with the reviewer and have replaced the word ‘dampening’ with ‘lower amplitude evening peak’ or similar wording throughout the manuscript*

Reviewer #3:

The study by Lamaze et al interrogates the behavioral function of the so-called evening clock neurons of the drosophila brain, as defined through their contribution to evening activity in in LD cycles, in temperature cycles with constant light (LLTC), where the evening activity is observed t the end of the thermophase. The data from cell ablation and clock stopping experiments support a contribution of the same cry-expressing pdf-negative lateral neurons to evening activity in these conditions, whereas PER rescue experiments indicate that they are not sufficient to produce evening activity. The authors conclude that these neurons are intrinsic evening cells, independently of the environmental conditions.

This is an interesting piece of work although I am not sure to understand why the authors emphasize the similarities of the behaviors in the two environmental conditions, since they conclude that the LD-defined E cells contribute to evening activity in LLTC but are not sufficient to produce evening activity in such conditions. I also believe that some experiments need to be reinforced to conclude about their requirement for evening activity, as detailed below.

1- p6 and Figures 1,2 + S1

The determination of the slope is not very clear to me. I might miss something but based on the formula, the ∆ activity/∆ time should be zero if there is no change in activity levels between the considered time points. In fig 1E, why is the mai179>cycDN ratio close to zero while the activity appears to strongly increase between the minimum value at ZT 7.5 and the peak value around ZT10 in fig 1C?

*The reviewer is correct and we now made an effort to explain better how we quantified the slope and synchronization of flies (see also response to point 2 of reviewer 1 above).*

*The steepness of the slope (Δactivity/Δt*) *describes the speed of anticipatory behavioural activity increase before the temperature transition in the evening. We calculate slope for each individual on the 6^th^ day of LLTC, which gives us a measure of the amplitude of anticipatory behaviour. These individual slope values are plotted as box plots throughout the manuscript. In order to define and at the same time quantify synchronization we now compare the slope of each individual to the theoretical slope calculated based on the median of the population (i.e. in Figure S1A: ∆ median activity (67.6)/∆ time (180 min) = 0.38) . The idea here is that if the individuals of a population are synchronized to each other, most individual slope values will be similar to the median, resulting in a ratio of median experimental to median theoretical slope close to 1. If the population is less well, or not synchronized, this ratio will be clearly below 1. These ratios are tabulated in the revised version of the manuscript or specified within the results text. Finally, we calculated the percentage of flies for each genotype with an experimental slope higher than half the value of the theoretical slope where a high versus low percentage again indicates synchronized or desynchronized behavior, respectively. These percentages are also plotted with the respective figures of the revised manuscript.*

*For the particular example mentioned by the reviewer (Mai179>cycDN in LLTC, Fig 1E), the box plot already shows the great variability of individual slope values (wide box, many outliers), giving rise to a median of individual slope values of 0. Therefore the ratio of experimental median (0) to theoretical median (0.17) is also 0 meaning the population is poorly synchronized. In fact, only 40% of all flies from this genotypes show a positive slope > half of the theoretical median, indicating poor synchronization.*

The authors conclude “demonstrating that a functional clock in the evening cells is necessary for synchronizing the evening activity peak in LLTC “.

As indicated above, the evening anticipation still appears robust in mai179>cycDN, although strongly reduced compared to controls. It is difficult to know whether the remaining anticipation is the consequence of the non-complete efficiency of clock-killing by cycDN or to the contribution of other clock neurons, but it seems to me that the synchronization still occurs. Stopping the clock with other tools might help to make the difference but I believe that the conclusion should be toned down with the current data.

*The reviewer is correct when only considering the anticipatory behaviour shown in Figure 1A. This is exactly the reason why we decided to use an alternative method of quantifying synchronized behaviour, which we extended in the revised version and also tried to explain more clearly. Our slope analysis and estimation of synchronized flies (described in detail in the response to point 1 of this reviewer above) allows us to classify a population of flies from a particular genotype as synchronised or not-synchronised with great confidence. Based on these analysis, Mai179>cycDN flies have a median slope of 0 and also exhibit a wide distribution of individual slope values (see Figure 1E, S1B), indicating that individuals are not synchronized to each other. This is also reflected by the low percentage of Mai179>cycDN flies with slope values > ½ of the median slope (Slp_th_) (Fig S1C). We are therefore confident that clock silencing in Mai179>cycDN flies was efficient and that indeed a functional clock in Mai179 cells is required for synchronisation to temperature cycles in constant light. Because we obtained essentially the same result with Mai179 > cycDN, Pdf-gal80 flies, we think that our conclusion that a functional clock within E-cells is required for synchronisation is valid, and we therefore decided to leave it in the manuscript.*

2- p7-8 and figures 4-5

Killing the spE-gal4-defined evening cells (cry+LNds+5th LNv) does not affect evening anticipation in LD (fig 4B). This challenges the role of the previous work blocking cry+ LNds+5thv with mai179-gal4/pdfgal80 and tetanus toxin (Johard 2009).
Since the ablation reported here does not seem to be complete, it would be important to reinforce this interesting result with another way of preventing these cells to function to allow such a conclusion.
In LLTC, SpEcells ablation is also much weaker than cry19, questioning the efficiency of the driver.
By the way, please add SpEgal4 to the methods paragraph and to table 1.

*The referee is correct that the SpE driver is weaker compared to Mai179 and cry19. We demonstrate (Fig S5D) and clearly state this on page 9 of the manuscript:*

‘To determine ablation efficiency we dissected *spE>hid,rpr* flies in LD at ZT2-ZT3 and immunostained the brains with PER as a marker. Although not 100% penetrant, in the majority of brains only 3 to 4 LNd could be detected, presumably representing the CRY- LNd subset. In addition, the 5^th^ s-LNv was detectable in 50% of the hemispheres, although it was never detectable in both hemispheres of the same brain (S5D Fig). Hence, the only partial desynchrony observed in LD, is most likely attributable to the not fully penetrant cell ablation.’

*Moreover, our improved analysis now reveals an impairment in LD synchronization, because both, the ratio of the experimental slope to the median slope, as well as the percentage of synchronized flies is reduced compared to controls (see Fig S5 of the revised manuscript). Our results therefore do not contradict previous reports (e.g. Johard et al 2009) but support them. Finally, we have added spE-gal4 to the Methods section and to Table 1.*

3- p9 and Figure S4

The authors show that stopping the clock in E cells does not prevent synchronization to T°C cycles in DD, although it clearly delays the phase of the activity bout. I believe that such conclusions have already been made in Busza et al 2007. This work should be cited and the present data should be discussed to emphasize what is new.

*The referee is correct and we apologize for not citing the Busza et al study in our original submission. Moreover, we now performed additional experiments restricting clock function to the morning neurons using both cry19-gal4 > cycDN and Mai179-gal4 >cycDN in combination with Pdf-Gal80 (Fig 6, S7). The results nicely show that the phase delay observed by the reviewer depends on cycDN expression in the morning neurons, because it is only observed in the flies not expressing Pdf-gal80. Our results are therefore in line with previous publications (Busza et al 2007, Gentile et al 2013) and we now refer to these studies when describing the DDTC results and elsewhere in the manuscript.*
