# Supplementary material for: Light triggers a network switch between circadian morning and evening oscillators controlling behaviour during daily temperature cycles
Source: PLoS Genet. 2022 Nov 11;18(11):e1010487. doi: 10.1371/journal.pgen.1010487 (PMC9683589; doi:10.1371/journal.pgen.1010487)
Supplement: Attachment — Submitted filename: reponse to reviewers August_15_ 2022_RS.docx [file pgen.1010487.s010.docx]

Reviewer #2: My first major concern was the confusion regarding the authors’ conclusion of “a more deterministic function” for E-oscillators.
The authors state that they have removed the term “deterministic” from their conclusions. However, this word is still used as the concluding sentence of the abstract in the revised manuscript. And, relating to my 4th major concern (see below) it is still not clear why such a function wasn't expected of neurons previously implicated as important for late day activity.

*We apologize, but we had used the wrong (old) version of the abstract in the revision by accident. The new abstract (which also addresses major point 4, see below) does not contain the word ‘deterministic’ anymore.*

My second major concern was over the apparent low penetrance of the cell ablation experiments. This is acknowledged by the authors in their response to the review. The survival of neurons targeted by ablation makes the results difficult to interpret cleanly.

*We agree with reviewer 2 that the low penetrance of the spE driver makes the results difficult to interpret. However, we decided to keep these data for two reasons. First, this driver is frequently used in the field without acknowledging or referring to its low penetrance. Hence, we believe that it is important to share this conclusion with the community: While being indeed specific for the E-cells, the penetrance of the spE driver is low.*

*Secondly, although the quantification of the PER+ LNd and 5^th^ in the spE>hid,rpr flies demonstrates a low penetrance of the gal4 driver, the behaviour data confirmed the necessity of the EO to synchronise the flies in LLTC. In LD the PDF neurons might play an important role in the robustness of the behaviour, because in LD the pdf>hid,rpr flies are not robustly synchronised while they are in LLTC.*

My third major concern was regarding the lack of a Mai-179-Gal4/Pdf-GAL80/UAS-CycDN. The authors have added this to their experiments, but, as pointed out by other reviewers, the results simply confirm previous work that M cells are important for rhythms under constant darkness.

*In the light of the model we are offering here (see response to major point 4 below), we think our results significantly extend previous findings showing that M cells are important for rhythms in constant darkness. They also go beyond the previous observation by Busza et al 2007 (J. Neuroscience 27: 10722) that M cells control the phase during constant darkness and TC. For example, Busza et al used a different temperature regime even though the amplitude of the temperature oscillation remains the same (20°C-29°C instead of the 25°C-16°C used here). Hence, although the molecular clock in the network should cycle the same way, the network itself could be at a different state because the absolute temperatures are different. Our model can also explain why the behavioural activity peak is at different time of day with different TC used (Gentile et al 2013, Curr Biol 5:e315). In different temperature conditions the balance within the network maybe different. For example, during DDTC29-20 the activity peak occurs in the mid/second half of the thermophase (Gentile et al., Busza et al) while in DDTC25-16 the peak occurs at the beginning of the thermophase (Gentile et al., Lamaze et al and this study). Busza et al found that removing the Pdf cells does not affect the behavioural phase under this warm TC (compare fig. 3B to A of Busza et al) however Pdf-ablated flies become AR in the following DD20°C. Removing the cry+ cells using cry[13]-gal4 (hence, different from the cry[19]-gal4 we are using in this study), largely abolishes both the evening peak in DDTC29-20 and the free running rhythm in the following DD20°C (Fig. 6B of Busza et al). Hence, we can conclude that in DDTC29-20 the CRY+ PDF- cells (that would include the E-cells) control the evening peak but not the free running rhythms in the following DD20°C. Note that in Gentile et al following DDTC29-20 the peak of Canton S flies in DD20 is advanced compared to the previous DDTC (Fig. 3B of Gentile et al), suggesting that under this environmental condition the network changes its balance (most likely because the temperature is different). Essentially the same phenomenon can be observed with the y w flies used by Busza et al. (e.g. Figs 3A, 6A of Busza et al). Here, what we observe is that a clock in the CRY+ PDF- cells is not necessary to synchronise and phase the behaviour to the morning in DDTC25-16 (Fig 5), while according to Busza et al a clock in the CRY+ PDF- neurons is sufficient to drive an evening peak in DDTC29-20 (Fig 6E of Busza et al). What can we conclude from this? Comparing these data would argue in favour of a change of the network balance when the environment changes. In the absence of light and a temperature of 25°C the balance switches toward a dominancy of the MO, while the evening cells rule at 29°C. The parallel presence of light and 25°C also switches the balance toward a dominancy of the EO.*

*It is interesting to note that whether in DD25-16 the MO clock is completely absent (Cry > cycDN, Fig 5) or just partially impaired (Mai179 > cycDN, Fig S7), flies present a peak delayed compared to controls. The strength of the clock disturbance determines the robustness of the peak. This suggests that another oscillator is under the influence of the MO. The best candidate being the DN1p (Chaterjee et al., 2018, Curr Biol 28:2007). Nonetheless, our data clearly demonstrate that under this environmental condition (no light 25°C) the EO is not in charge while it becomes in charge in the presence of light although the temperature remains the same. We added these arguments to our discussion.*

My fourth concern was that the authors did not present alternative model of clock network function based on these new results, nor did they provide a clear or compelling revision in our conception of the network or how it entrains to distinct environmental rhythms.

*Serge Daan said during the 6^th^ meeting of SRBR :”Light intensity varies as much between sunny and cloudy days as it does in the average curve over the time…This poses the true dilemma for entrainment. On one hand, these circadian systems should be sensitive toward relatively small changes in light intensity. On the other hand, they can only exploit these changes as averaged over many days rather than respond to each cloud that hides the sun”. (Serge Daan 2000, J Biol Rhythms 15, 195). He presented here the true remaining enigma of the circadian clock field. We believe that we need to extend the models developed by the circadian theorists Colin Pittendrigh (non parametric effect of light entrainment), Jürgen Aschoff (parametric effect of light entrainment) and Serge Daan himself who tried to combine both models into one. In the light of the current knowledge, we propose a model where the clock network formed of multiple oscillators is entrained in a robust manner to Zeitgebers, with light and temperature being on/going up and off/down once a day each. On the other hand environmental inputs such as light and temperature (but not exclusively), are integrated by the network itself modulating the balance of interactions within that network and leading to an adaptation of the timing of the peak (phase) of locomotor activity.*

*Hence, we only offer here the base on which to build and demonstrate this model experimentally. The next step can be for example to understand how light-input is integrated by the network to shut down the MO, while the EO is active. The same can be done vice versa for temperature. Concerning temperature, a candidate are the DN1p and their interaction with the trpA1-expressing AC neurons, because trpA1 is not involved in temperature entrainment per se (Roessingh et al 2015, J Biol Rhythms 30: 492). This interaction may explain the temperature dependent phase changes observed between DDTC25-16 and DDTC29-20 (see discussion).*

*Therefore, agreeing with reviewer 2, we believe that the originality of our work is not about the EO driving an evening peak. It is about developing a new model to explain the inconsistencies of circadian robustness and clock-output plasticity. Our work may also inspire theoretical biologists and modellers to integrate our experimental findings in extended theoretical entrainment frameworks. To make our model more easily understandable for the reader we generated a new figure (Fig 6 of the revised manuscript) explaining our model in form of a cartoon.*

The authors have still not sufficiently explained why the finding that the evening cells make a significant contribution to evening activity under LLTC cycles is unexpected. The authors do state that this is surprising based on previous work by Yoshii and colleagues (2010) showing that Cry- neurons have molecular clocks that are more strongly driven by temperature cycles than Cry+ neurons. They also showed that Cry- neurons were sufficient for (bimodal) rhythms under light/moonlight plus temperature cycles and that flies lacking Cry+ neurons did not re-entrain to a shifted temperature cycle when the Light-Moonlight cycle was kept at the same phase. However, it does not logically follow that this means E cells would not be expected to make significant contributions to the evening peak of activity under LLTC, if present and intact within the network. It is therefore still not clear to me why the major results are not expected. E cells drive evening activity under entrainment. Why would we have predicted otherwise?

*We agree with the reviewer 2 that it is not unexpected. We do confirm that CRY-negative neurons can independently be synchronized to temperature cycles (if the clock in the Cry-positive neurons is stopped, the CRY-negative neurons are still synchronized to temperature cycles (Fig 2). Furthermore, we recently showed that PER and TIM cycling can be synchronized in the absence of CRY, although with a different phase compared to wild type flies (Lamaze et al 2022, Nat Commun 13:1724). Nevertheless, as we show here, clock function in the CRY-negative neurons does not mediate behavioural activity in LLTC, which if not surprising, we think it is at least a very important finding. Hence, the ‘Yoshi model’ of independent temperature and light oscillators should be revisited in light of our current observation. Because the focus of our study is on activity phase (determined by an interplay of the clock and the environmental condition), and not on temperature entrainment as such, we only briefly discuss this issue at the end of our revised discussion.*

Reviewer #3: The following is still not clear: a) Why has synchronisation among individuals been used and is it is the same as inter-individual variation? And what can this tell us about the role of these cells under LLTC. b) The rationale of using this measure over traditional measures of anticipation index or peak timing is still unclear. c) While to some extent the calculations are now clear it is still difficult to understand why this measurement of using slope(theoretical) value was obtained.

Why was the median used for plotting the activity profiles instead of mean? Was the data skewed. No error was reported (as previously pointed out). Since error around median does not always make sense, often range (minimum to maximum) is reported. If the slope ratio was calculated using median values what sort of statistical test was used to make the comparisons shown in Figure1 D and E. The tests have not been explicitly mentioned in the methods section.

*a-c) We agree with reviewer 3, the higher the synchronisation is, the lower is the inter-individual variability. Hence, we removed the term “between individuals” and rewrote the description of the method.*

*P6: “*A decrease in amplitude can be due to reduced synchronization within the population, or to reduced speed of activity increase resulting in a delayed activity peak. To first quantify the amplitude reduction, we calculated for each fly their slope (the speed of activity increase, Δactivity/Δt). We determine the two time points based on the median level (S1A Fig and Materials and Methods). Using this method, we observed a strong decrease of the slope in both *Mai179>cyc^DN^* and *cry[19]>cyc^DN^* flies in LD and LLTC (Figs 1D, E, S1B). To test whether this decrease was due to a decrease of synchronization or a (synchronized) delay of anticipatory activity, we compared the median of the slope value (Slo_exp_) with the slope of the median (Slo_the_). Therefore, if fly activity is highly synchronized between individuals, the ratio of Slo_exp_/Slo_the_ is close to 1 (i.e., most individuals behave similar to the median). While the Slo_exp_/Slo_the_ ratio for the controls was close to 1, it was reduced to ~0.5 for both *Mai179>cyc^DN^* and *cry[19]>cyc^DN^* flies (S1B Fig). In addition, to help visualizing if a population is synchronized, we compared the percentage of flies that have a Slp_exp_ > ½ x Slp_the_ with flies that are under that value.”

*b-c) Many methods have been used by different groups, including ours, however we developed a novel one that more accurately shows (1) if there is a peak, (2) if it is robust (synchronised) or delayed (low amplitude but robust). The median of the normalised activity is more powerful than the average of the activity and somewhat better than average of the normalised activity as it is more representative of the synchronisation of the population. Just by looking at the graph, the closer the median value is to 100, the more synchronised the population is. The experimental slope (the population of individual slope values) tells us how fast flies are increasing their locomotion. And if that value is close to the slp_th_ (how fast the median is increasing) then the flies are synchronised. A lower amplitude of the median (<50) could be due to a low synchronisation or because the peak is delayed. If the synchronisation is reduced then comparing the median of the slope with the slope of the median will indicate how synchronised a population is.*

*Normalising the activity removes the variability of activity level, which is most likely due to different genomic background*

*The median of the normalised activity is more representative of the synchronisation. Putting the interquartiles in the graphs would have been too messy as we would not have been able to compare in the same graph the experimental with its parental controls. However, the ratio slo_exp_/slo_the_ as well as the box plot of the population of slopes and the percentage of flies anticipating are strongly complementing the graphs.*

*Comparisons between panel D and E in Figure 1 have been done using Kruskal Wallis tests (non parametric for comparing medians) as mentioned in the Figure legend.*

• “Our data indicate that morning oscillator neurons dictate morning activity during DDTC (31), while evening neurons are required in LLTC for setting the activity phase to the afternoon (Fig 1).”

• An alternate view could be that under constant temperature conditions, M cells are the pacemakers in DD and E cells are the pacemakers under LL (at least when CRY is absent). Neither of these cell types are reported to express temperature sensors. Thus, temperature information must be relayed to them either from outside the clock circuit or from other clock neurons possessing temperature sensors like DN1p as mentioned in the paragraph below

*We agree with reviewer 3 however, we are not sure how this differs from our interpretation. Also, to our knowledge, the DN1p do not possess temperature sensors either. However, they receive warm temperature input from the AC neurons (trpA1[SH] and ppk-gal4) (Lamaze et al 2017, Sci Rep 7: 40304) as well as inputs from peripheral thermos sensors (Chen et al 2018, Curr Biol 28:1725; Yadlapalli et al 2018, Nature 555: 98).Nonetheless trpA1 is fully activated by temperature >25°C. Here we are using 25°C-16°C. However, this is a good point that could explain the phase difference in DDTC29°C-20°C where flies present a delayed peak compared to 25°C-16°C (see discussion with reviewer 2). Perhaps there, the DN1p play a more prominent role in phasing the behaviour as they receive temperature input from the trpA1 activated neurons. We now discuss this possibility in the revised version of our Discussion.*
