# Supplementary material for: Light triggers a network switch between circadian morning and evening oscillators controlling behaviour during daily temperature cycles
Source: PLoS Genet. 2022 Nov 11;18(11):e1010487. doi: 10.1371/journal.pgen.1010487 (PMC9683589; doi:10.1371/journal.pgen.1010487)
Supplement: Attachment — Submitted filename: response to reviwers.docx [file pgen.1010487.s011.docx]

Reviewer #2: The authors have added a model to the revised manuscript in response to my persistent concerns regarding how the work produces a change in our understanding of circadian timekeeping, but this model lacks sufficient mechanistic detail to be useful to the field. The authors have also replaced the original abstract that still contained the conclusion regarding the work supporting a "more deterministic" function for E oscillators. These are improvements. But my concerns remain, notwithstanding the elaborate discussion of previous work in the authors' response to the review. The results described in the manuscript largely support previous work indicating that the balance of control shifts within the clock neuron circuitry as a function of environmental conditions. This manuscript covers familiar ground in this regard: M cells dominate in darkness and non M cells dominate in the light. I am still not convinced that the manuscript significantly changes our conception of the network in a clear or compelling way.

*We thank reviewer 2 for sharing his/her doubts about the novelty of our work. Although we thought to have addressed all remaining concerns, it appears that an important misunderstanding remains. The reviewer is correct that in light:dark (LD) cycles, the EO drives the evening peak while the MO drives the morning peak. In contrast, our work aims to understand how a certain environmental information ‘A’ (‘A’ being the light in our experimental paradigm) is integrated by the circadian system to time (phase) the behavioural response, while the molecular clock is synchronised to a distinct external environmental factor ‘B’ (‘B’ being temperature in our experimental paradigm). This is an important biological question, because in nature sudden environmental changes of one cue (e.g., temporary drop or increase of temperature)* ***must not*** *be interpreted as Zeitgeber entraining the circadian clock, although it* ***must*** *elicit a behavioural response (e.g., seeking shade or sun, increase or decrease activity). Here, we are offering a template for a new model explaining how the circadian system can solve this problem and we present how it can be tested experimentally (see author summary). Because we used temperature cycles (TC) as the sole Zeitgeber we demonstrate that light uses distinct pathways for the entrainment of the molecular clock and for controlling the timing of the circadian output (phase), via modifying the neuronal network balance. By supplying strong experimental evidence for our model with the work presented here, we and others will now be able to dissect the molecular and neuronal mechanisms enabling this biologically important distinction.*

*The results presented here support our model, because we show that while daily TC robustly entrain the different neuronal oscillators constituting the circadian network (cue ‘B’ above), light promotes the output of the EO and darkness the output of the MO (light or dark representing cue ‘A’). Although instinctively not surprising, this is important to state in order to establish the base for new studies, because we demonstrate that Zeitgeber and modulator of the neuronal network function of light can be distinguished. By supplying strong experimental evidence for our model with the work presented here, we will be able in the future to dissect the molecular and neuronal mechanisms enabling this biologically important distinction.*

*To make the aim of our study and the model we propose and test here clearer, we rewrote the 2^nd^ part of our abstract and parts of the Discussion. We hope that the importance and novelty of our study is now clearer for the reviewer and the readers of PLoS Genetics.*
